# Clinical infection of *Brucella canis* in a companion dog with discospondylitis in the Republic of Korea

**DOI:** 10.17221/37/2023-VETMED

**Published:** 2023-07-28

**Authors:** Ju-Hee Seo, Ye-In Oh, Se-Hoon Kim, Kyoung-Won Seo, Byung-Jae Kang

**Affiliations:** ^1^Department of Veterinary Clinical Sciences, College of Veterinary Medicine and Research Institute for Veterinary Science, Seoul National University, Seoul, Republic of Korea; ^2^Department of Veterinary Internal Medicine, College of Veterinary Medicine, Kyungpook National University, Daegu, Republic of Korea; ^3^Department of Veterinary Internal Medicine, College of Veterinary Medicine, Seoul National University, Seoul, Republic of Korea; ^4^BK21 FOUR Future Veterinary Medicine Leading Education and Research Center, Seoul National University, Seoul, Republic of Korea; Ju-Hee Seo and Ye-In Oh contributed equally to this work

**Keywords:** *Brucella canis*, discospondylitis, doxycycline, enrofloxacin, zoonotic disease

## Abstract

A 2-year-old, spayed female, Bichon Frise dog was presented with reluctance to exercise, back pain, and frequent sitting down. Multiple osteolysis, periosteal proliferation, and sclerosis of the vertebral endplates of T11–13 were observed in the radiography, computed tomography, and magnetic resonance imaging. The bacterial culture of the urine specimen, the polymerase chain reaction (PCR) of the blood, and the antibody tests were positive for *Brucella canis*. Accordingly, discospondylitis caused by *B. canis* was diagnosed and doxycycline was administered. The clinical signs resolved and the culture and PCR results were negative afterwards. Doxycycline was discontinued after 6 months. The clinical signs recurred 2 weeks later, and the combination treatment of doxycycline and enrofloxacin was initiated. Though no clinical signs were observed after 9 months and the bacterial cultures and PCR were negative, the antibody titre remained at 1 : 200 or more. The dog will continue taking antibiotics until the antibody titre drops. To the best of our knowledge, this is the first case report of a clinical infection of *B. canis* associated with canine discospondylitis in the Republic of Korea. Although the clinical signs of brucellosis might improve with antibiotic treatment, the disease cannot be cured due to *Brucella*’s various strategies to evade host immune systems. Specifically, it can proliferate and replicate within the host cells, resulting in an environment that makes treatment less effective. Furthermore, owing to its zoonotic potential, owners and veterinarians should consider lifelong management or euthanasia.

Discospondylitis is an inflammation of the intervertebral discs associated with the end plates and nearby vertebral bodies. Several organisms can cause discospondylitis. The most common pathogen is *Staphylococcus* spp. and although *Brucella canis* is uncommon, it has also been detected occasionally (Moore 1992; Thomas 2000). The haematogenous spread to the disc region is the most typical infection pathway. Dogs with discospondylitis display various clinical signs such as depression, anorexia, reluctance to move, and neurologic dysfunctions (Kornegay and Barber 1980; Moore 1992; Thomas 2000). The radiographic signs of discospondylitis are the irregularity and erosion of the end plates and the lysis of the nearby bones. Subsequently, bone proliferation and sclerosis occur (Kornegay and Barber 1980; Jimenez and O’Callaghan 1995; Thomas 2000). Discospondylitis is generally confirmed through radiography. The presumed causative organism is typically identified through blood and urine cultures*.* Owing to *Brucella’s* zoonotic potential, at minimum, a *Brucella* serological test should be performed so that biosafety measures can be implemented (Santos et al. 2021).

*B. canis* is the most common brucellae isolated from dogs. In dogs, *B. canis* mainly causes reproductive disorders in sexually intact animals; however, it can also cause discospondylitis regardless of sexual status (Thomas 2000). There are various diagnostic methods for canine brucellosis, including serological tests such as the rapid slide-agglutination test (RSAT), 2-mercaptoethanol RSAT (2ME-RSAT), agar gel immunodiffusion (AGID), and enzyme-linked immunosorbent assay (ELISA); blood and urine bacterial culture; and molecular diagnostic methods such as polymerase chain reaction (PCR). Since the existing serological diagnostic methods have low specificity (i.e., may have false positive results), confirmatory tests such as a culture or PCR must also be performed (Wanke 2004; Mol et al. 2020). Direct exposure to bodily fluids from dogs infected with *B.* *canis* can lead to human infection. Oronasal exposure is the most common route (Santos et al. 2021). People most often experience fatigue, relapsing fever, and weight loss as clinical signs (Young 1995; Sayan et al. 2011; Santos et al. 2021). However, severe conditions, such as endocarditis, arthritis, and osteomyelitis, have been described in human patients as well (Tosi and Nelson 1982; Ying et al. 1999; Marzetti et al. 2013; Santos et al. 2021).

On entering the animal’s body, *Brucellae* are phago-cytised by cells like macrophages, then move to the lymph nodes and circulate in the blood throughout the body, causing clinical signs (Wanke 2004). *Brucella* is an intracellular bacterium with no curative treatment protocol and a high recurrence rate. There is no commercially available vaccine for the prevention of canine brucellosis.

We describe the first officially reported case of identifying and managing a *B. canis* infection in a companion dog diagnosed with discospondylitis in the Republic of Korea.

## Case presentation

**Figure 1:**
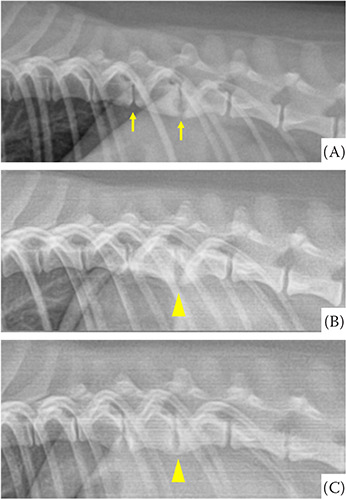
Right lateral radiographs of the thoracic spine (A) at presentation, (B) 6 months, and (C) 15 months after antibiotic administration Local bone lysis, proliferation, and sclerosis are found on the T11–13 vertebral end plates (arrows). Smoothly marginated and osseous infilling of T12–13 vertebral endplates (arrowhead) over time

A 2-year-old, spayed female, Bichon Frise dog was presented with reluctance to exercise, pain response when picked up by the owner, and frequent sitting down while walking. The dog was previously administered amoxicillin/clavulanate acid and cephalosporins for a long time to treat the recurrent cystitis. The physical examination showed increased muscle tone and back pain at the thoracolumbar junction. There were no neurologic deficits or any specific abnormalities detected during the orthopaedic examination. Both the serum biochemistry panel and complete blood count revealed no abnormalities. Radiographs showed a ventral spur formation between the T12–13 vertebral body, local bone lysis, and sclerosis between the T11–13 vertebral end plates (Figure 1).

**Figure 2:**
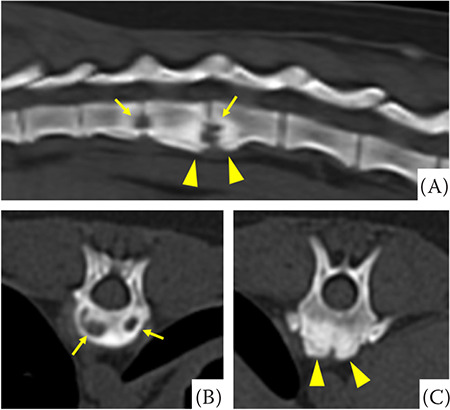
Sagittal (A) and transverse planes (B, C) of bone window computed tomography (CT) at presentation Osteolysis (arrows) and proliferation (arrowheads) with sclerosis of the T11–13 vertebral endplates can be seen

No abnormalities were detected on the thoracic and abdominal radiographs and the abdominal ultrasound. The computed tomography (CT) showed multiple bone lysis between the T11–13 vertebral end plates and bone proliferation between the T11–13 vertebral bodies (Figure 2).

**Figure 3:**
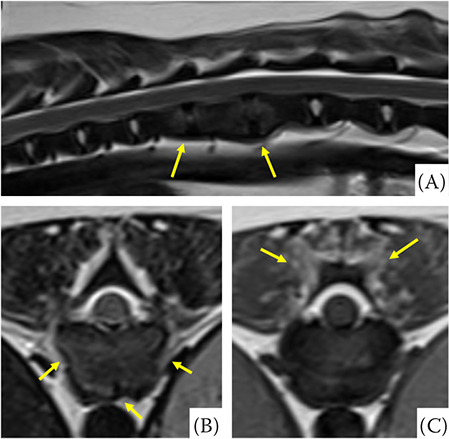
Sagittal (A) and transverse planes (B, C) of the T2 W images in the magnetic resonance imaging (MRI) at presentation (A) The hyperintensity of the T11–13 vertebral endplates (arrows). (B) The irregularity margin of the end plate (arrows). (C) The hyperintensity of the paraspinal soft tissues around the lesion (arrows)

The magnetic resonance imaging (MRI) showed loss of shape and normal hyperintensity of the discs between T11–13 in the T1 W images. The irregular margin and hyperintensity of the T11–13 vertebral endplates and hyperintensity of the paravertebral soft tissue around T12 were identified in the T2 W images (Figure 3).

A blood sample was aseptically obtained, and a urine sample was obtained through cystocentesis. To identify the pathogens of discospondylitis, the *B.* *canis* indirect fluorescent antibody (IFA) test (IDEXX Laboratories, Westbrook, ME, USA) and an aerobic and anaerobic blood (POBANILAB, Namyangju, Republic of Korea) and a urine bacterial culture (Neodin Biovet laboratory, Guri, Republic of Korea) were performed. A few days later, the pathogen suspected to be *Brucella* was cultured from the aerobic bacterial urine culture. Due to the risk of a zoonotic disease, the dog was reported to the Animal and Plant Quarantine Agency (APQA) of the Republic of Korea. The serological tests [2ME-RSAT, immunochromatography test (ICT)] and bacterial culture and PCR for *B.* *canis* of the blood samples were performed by the APQA and all the tests were positive for *B. canis*. The *B.* *canis* IFA test was positive at over 1 : 200 (Table 1).

**Table 1:** Bacteriologic, serologic and polymerase chain reaction (PCR) results during the treatment period in this case

Month after treatment	Blood culture	Urine culture	Blood PCR	IFA	2ME-RSAT	ICT
0	+	+	+	≥ 1 : 200	+	+
2	–	–	–	≥ 1 : 200	+	+
4	–	–	–	≥ 1 : 200	+	+
6	–	–	–	≥ 1 : 200	+	+
9	NA	NA	–	≥ 1 : 200	NA	NA
12	NA	NA	–	≥ 1 : 200	NA	NA
15	NA	NA	–	≥ 1 : 200	NA	NA

(–) = negative; (+) = positive; 2ME-RSAT = 2-mercaptoethanol rapid slide-agglutination; ICT = immunochromatography test; IFA = indirect fluorescent antibody; NA = not assessed

Based on the above results, the dog was diagnosed with discospondylitis due to *B. canis* infection. The antibiotic sensitivity tests (Neodin Biovet laboratory, Guri, Republic of Korea) showed that this pathogen was sensitive to doxycycline and moderately sensitive to enrofloxacin.

The antibiotic therapy was initiated using doxycycline (10 mg/kg, p.o., q24 h) according to the antibiotic sensitivity test. The owner agreed to continue to monitor the dog and quarantine the dog at home.

Two months after taking antibiotics, a blood culture, urine culture, and *B. canis* IFA test were performed. In APQA, the 2ME-RSAT, ICT, and *Brucella* PCR of the blood samples were re-examined. The *B. canis* IFA test showed higher than 1 : 200; however, the blood and urine culture tests were negative. The 2ME-RSAT and ICT were positive; however, the PCR was negative (Table 1). The dose of doxycycline was increased to 15 mg/kg (p.o., q24 h) in the second month of treatment. Follow-up examinations were conducted two months and four months after the dose escalation. The *B. canis* antibody titre was still high and the 2ME-RSAT and ICT were positive. However, the blood culture, urine culture, and *B. canis* PCR were negative (Table 1).

The radiography showed mild radiographic improvement with the bone proliferation replaced lytic lesions (Ruoff et al. 2018) (Figure 1). Considering that the clinical signs resolved for six months, and the bacterial cultures were negative, the antibiotic was discontinued.

Back pain recurred after two weeks of the discontinuation of doxycycline; therefore, a combination of enrofloxacin (10 mg/kg, p.o., q24 h) and doxycycline (10 mg/kg, p.o., q12 h) were prescribed. The pain response significantly decreased a few days later, and antibiotics were continuously administered. Three months later, a *B. canis* PCR of the blood sample and a *B. canis* IFA test were performed, and the PCR was negative; however, the IFA test was higher than 1 : 200 (Table 1). There were no clinical signs for the next nine months and the radiography showed a marked radiographic improvement with smoothing and the loss of the lytic lesions (Ruoff et al. 2018) (Figure 1). In the blood and urine cultures, pathogens were not detected. Since, in the *B. canis* IFA test, the antibody titre was still high, we decided to continue the antibiotics until the antibody titre was lower than 1 : 50 because it typically suggested previous exposure to the *Brucella* bacteria; however, it did not necessarily represent an ongoing infection.

The patient has been on antibiotic therapy for 15 months since the diagnosis and remains asymptomatic; however, the antibody titre remains elevated at > 1 : 200, indicating that the brucellosis has not been cured even though the clinical signs are controlled.

## DISCUSSION

The most prevalent cause of canine brucellosis is *B. canis.* Since *Brucella* is an intracellular bacterium, it is difficult to treat and has an extremely high recurrence rate (Wanke 2004). Additionally, owing to the possibility of transmission to humans, it is designated as a legally notifiable infectious disease in the Republic of Korea. In Japan, the 2020 epidemiological survey showed that 2.9% of dogs were serologically confirmed with *Brucella*, of which 3.6% were stray dogs, and 7.9% were in shelters (Kei et al. 2020). In 2019, a study on the prevalence of *B. canis* at a breeding kennel in Ontario, Canada, showed seropositivity in 127/1 080 cases (11.8%) (Weese et al. 2020). According to a survey of *B. canis* in Michigan, USA between 2007 and 2016, seropositivity rates for purebred dogs possessed by non-commercial breeders were 0.4%, and production facilities ranged from 2/22 (9%) to 5/6 (83%) (Johnson et al. 2018). In the Republic of Korea, 2 394 dogs (1 825 companions and 569 strays) were surveyed in 2015–2016, 16 (0.9%) of the 30 positive dogs were companion dogs and 14 (2.5%) were stray dogs (Jung et al. 2018). Previously, *Brucella* infections were mostly reported in stray dogs or kennel dogs; however, these findings show that non-breeding or neutered companion dogs can be infected with *Brucella*. As the pet industry grows rapidly, appropriate management methods for brucellosis are needed.

Brucellosis is difficult to treat, and a curative antibiotic treatment regimen has not yet been found. Euthanasia can be proposed owing to the epidemic characteristic; however, guidelines for companion animals are ambiguous. In other countries, such as the United States or Canada, when *Brucella* infection occurs in companion dogs, long-term antibiotic treatment is often considered rather than euthanasia (Cosford 2018; Hensel et al. 2018) if the owner agrees to quarantine and continuously monitor the dog. Many attempts to treat *Brucella* infections have been reported. *In vitro*, enrofloxacin and streptomycin were synergistic, and doxycycline and rifampicin were antagonistic in certain strains of *B. canis* (Mateu-de-Antonio and Martin 1995). In 10 females and 2 males, enrofloxacin (5 mg/kg, p.o., q12 h) was administered for 30 days, and 9 females took additional enrofloxacin during the oestrus stage. The RSAT results of 10 dogs were negative after 14 months and the offspring born to the female dogs who had been infected and treated were healthy (Wanke et al. 2006). In dogs with endophthalmitis caused by *B. canis*, enrofloxacin (10 mg/kg, p.o., q24 h); doxycycline (15 mg/kg, p.o., q12 h); streptomycin (20 mg/kg, i.m., q24 h for 7 days, every other week for 8 weeks); and rifampin (7.5 mg/kg, p.o., q24 h, after streptomycin is completed) have been used to treat brucellosis. *B. canis* could not be cultured from the blood samples and attained a seronegative status after a median of 96 weeks (range: 36–112 weeks) of treatment (Ledbetter et al. 2009).

While selecting antibiotics for the patient in our study, we considered the results of the antibiotic sensitivity test, the methods of taking drugs, and the side effects. Doxycycline and enrofloxacin were selected because both showed susceptibility to the bacteria, could be taken orally, and had minimal side effects. During the use of antibiotics, there were no clinical signs and the bacterial cultures and *B. canis* PCR were continuously negative; however, the serological antibody titre was consistently high. This suggests that the clinical signs might be managed in brucellosis; however, it is difficult to cure completely. Antibody titre may be positive if the infection is still present or if previously produced antibodies are present. In one study, three patients infected with *B. canis* were negative for both the culture and serological antibody tests, and the median value for that treatment period was 96 weeks (range 36–112 weeks) (Ledbetter et al. 2009). Therefore, a long-term follow-up is needed because the seronegative result takes a very long time to appear. In the USA, 100 to 200 occurrences of human brucellosis (caused by all *Brucella* species) are reported each year (Cosford 2018). *B. canis* accounts for only 1% of the confirmed cases of human brucellosis; however, its stealthy nature in comparison to pathogenic smooth *Brucella* can contribute to the underdiagnosis (Santos et al. 2021). Thus, owners and veterinarians should remain vigilant for any potential disease transmission during the prolonged treatment of an infected dog.

Another problem is that there may be side effects from long-term antibiotics. The side effects of long-term doxycycline and enrofloxacin use are poorly understood. An increase in liver enzyme activities and gastrointestinal signs were the most common side effects of doxycycline therapy. In particular, the alkaline phosphatase (ALP) activity is associated with the dose of doxycycline (Schulz et al. 2011). The most frequent adverse effects of enrofloxacin are digestive issues like nausea, abdominal pain, vomiting, and diarrhoea (Trouchon and Lefebvre 2016). In this study, the dog was given famotidine (0.5 mg/kg, p.o., q12 h), gastrointestinal protection, and silymarin (10 mg/kg, p.o., q12 h), a liver supplement, and gastrointestinal problems such as vomiting and diarrhoea have not yet appeared. Doxycycline and enrofloxacin have been administered together for nine months and are well tolerated by the patient.

To the best of our knowledge, this is the first case report of managing a clinical infection of *B. canis* associated with canine discospondylitis in the Republic of Korea. While controlling the clinical signs of brucellosis may not be difficult, curing the disease is very challenging. It requires long-term treatment, is expensive to treat, and can easily reoccur. Therefore, owners and veterinarians must consider this aspect and decide the optimal approach for treatment. In addition, a consensus on the management of canine brucellosis is needed in the Republic of Korea.

## References

[R1] Cosford KL. Brucella canis: An update on research and clinical management. Can Vet J. 2018 Jan;59(1):74-81. 29302106 PMC5731389

[R2] Hensel ME, Negron M, Arenas-Gamboa AM. Brucellosis in dogs and public health risk. Emerg Infect Dis. 2018 Aug;24(8):1401-6. 30014831 10.3201/eid2408.171171 PMC6056133

[R3] Jimenez MM, O’Callaghan MW. Vertebral physitis: A radiographic diagnosis to be separated from discospondylitis: A preliminary report. Vet Radiol Ultras. 1995 May 1;36(3):188-95.

[R4] Johnson CA, Carter TD, Dunn JR, Baer SR, Schalow MM, Bellay YM, Guerra MA, Frank NA. Investigation and characterization of Brucella canis infections in pet-quality dogs and associated human exposures during a 2007–2016 outbreak in Michigan. J Am Vet Med Assoc. 2018 Aug 1;253(3):322-36. 30020006 10.2460/javma.253.3.322 PMC6642745

[R5] Jung JY, Yoon SS, Lee SH, Park JW, Lee JJ, Her M, So BJ, Kim JH. Prevalence state of canine brucellosis in South Korea during 2015 and 2016. Korean J Vet Res. 2018;58(3):125-9.

[R6] Kei N, Shingo S, Hidenori K, Soichi M. Seroepidemiological survey of Brucella canis infection in dogs in Japan. Jpn J Vet Res. 2020 May 1;68(2):129-32.

[R7] Kornegay JN, Barber DL. Diskospondylitis in dogs. J Am Vet Med Assoc. 1980 Aug 15;177(4):337-41. 7451303

[R8] Ledbetter EC, Landry MP, Stokol T, Kern TJ, Messick JB. Brucella canis endophthalmitis in 3 dogs: Clinical features, diagnosis, and treatment. Vet Ophthalmol. 2009 May-Jun;12(3):183-91. 19392878 10.1111/j.1463-5224.2009.00690.x

[R9] Marzetti S, Carranza C, Roncallo M, Escobar GI, Lucero NE. Recent trends in human Brucella canis infection. Comp Immunol Microbiol Infect Dis. 2013 Jan;36(1):55-61. 23040615 10.1016/j.cimid.2012.09.002

[R10] Mateu-de-Antonio EM, Martin M. In vitro efficacy of several antimicrobial combinations against Brucella canis and Brucella melitensis strains isolated from dogs. Vet Microbiol. 1995 Jun;45(1):1-10. 7653024 10.1016/0378-1135(94)00122-d

[R11] Mol JPS, Guedes ACB, Eckstein C, Quintal APN, Souza TD, Mathias LA, Haddad JPA, Paixao TA, Santos RL. Diagnosis of canine brucellosis: Comparison of various serologic tests and PCR. J Vet Diagn Invest. 2020 Jan;32(1):77-86. 31752635 10.1177/1040638719891083 PMC7003229

[R12] Moore MP. Discospondylitis. Vet Clin North Am Small Anim Pract. 1992 Jul;22(4):1027-34. 1641915 10.1016/s0195-5616(92)50091-2

[R13] Ruoff CM, Kerwin SC, Taylor AR. Diagnostic imaging of discospondylitis. Vet Clin North Am Small Anim Pract. 2018 Jan;48(1):85-94. 28964545 10.1016/j.cvsm.2017.08.007

[R14] Santos RL, Souza TD, Mol JPS, Eckstein C, Paixao TA. Canine brucellosis: An update. Front Vet Sci. 2021 Mar 2;8:594291. 33738302 10.3389/fvets.2021.594291 PMC7962550

[R15] Sayan M, Erdenlig S, Stack J, Kilic S, Guducuoglu H, Aksoy Y, Baklan A, Etiler N. A serological diagnostic survey for Brucella canis infection in Turkish patients with brucellosis-like symptoms. Jpn J Infect Dis. 2011;64(6):516-9. 22116333

[R16] Schulz BS, Hupfauer S, Ammer H, Sauter-Louis C, Hartmann K. Suspected side effects of doxycycline use in dogs – A retrospective study of 386 cases. Vet Rec. 2011 Aug 27;169(9):229. 21791480 10.1136/vr.d4344

[R17] Thomas WB. Diskospondylitis and other vertebral infections. Vet Clin North Am Small Anim Pract. 2000 Jan;30(1):169-82. 10680214 10.1016/s0195-5616(00)50008-4

[R18] Tosi MF, Nelson TJ. Brucella canis infection in a 17-month-old child successfully treated with moxalactam. J Pediatr. 1982 Nov;101(5):725-7. 6215476 10.1016/s0022-3476(82)80301-6

[R19] Trouchon T, Lefebvre S. A review of enrofloxacin for veterinary use. Open J Vet Med. 2016 Feb 26;6(2):40-58.

[R20] Wanke MM, Delpino MV, Baldi PC. Use of enrofloxacin in the treatment of canine brucellosis in a dog kennel (clinical trial). Theriogenology. 2006 Oct;66(6-7):1573-8. 16476476 10.1016/j.theriogenology.2006.01.034

[R21] Wanke MM. Canine brucellosis. Anim Reprod Sci. 2004 Jul;82-83:195-207. 15271453 10.1016/j.anireprosci.2004.05.005

[R22] Weese JS, Hrinivich K, Anderson MEC. Brucella canis in commercial dog breeding kennels, Ontario, Canada. Emerg Infect Dis. 2020 Dec;26(12):3079-80. 33219799 10.3201/eid2612.201144 PMC7706960

[R23] Ying W, Nguyen MQ, Jahre JA. Brucella canis endocarditis: Case report. Clin Infect Dis. 1999 Dec;29(6):1593-4. 10585833 10.1086/313545

[R24] Young EJ. An overview of human brucellosis. Clin Infect Dis. 1995 Aug;21(2):283-9; quiz 290. 8562733 10.1093/clinids/21.2.283

